# Transcriptome analysis of childhood Guillain–Barré syndrome associated with supportive care

**DOI:** 10.3389/fped.2022.1008996

**Published:** 2022-09-26

**Authors:** Ke Hu, Wanli Liu, Yi Gan, Zhaoxuan Huang

**Affiliations:** Department of Pediatric, Maternal and Child Health Hospital of Hubei Province, Tongji Medical College, Huazhong University of Science and Technology, Wuhan, China

**Keywords:** Guillain–Barré syndrome, pediatrics, transcriptome, nursing, biomarkers

## Abstract

Childhood Guillain–Barré syndrome (GBS) is a rare neurological disease. Early diagnosis followed by precise treatment can reduce mortality. In this study, we collected two transcriptome data between GBS and controls from the publicly available databases (GEO dataset). We identified two distinct down-regulated genes (PTGDS and AR) in GBS by transcriptome analysis (*n* = 20). Based on the two distinct down-regulated genes in the GBS group, a two-gene diagnostic signature was developed. Moreover, gene expression analysis for the two-gene was performed on a patient with GBS before and after Supportive Care. RT–PCR results show that the expression of PTGDS increased after the patient was given supportive care. Therefore, PTGDS might be considered as a potential target for therapeutic target in GBS.

## Introduction

The Guillain–Barré syndrome is a common cause of acute flaccid paralysis in children. A small number of children will develop respiratory muscle weakness, which is a self-limiting disease with a good prognosis (1). There are approximately 2 in 100,000 new cases of the Guillain–Barré syndrome each year, and the disease has a global mortality rate of about 7.5% (2). It is typically accompanied by allodynia and muscle weakness, usually beginning with the hands and feet, and then moving to the arms and upper body (3). During the acute phase of the disease, 15% of patients will develop respiratory muscle attacks that may be life-threatening and require mechanical ventilation (4). Some may affect the autonomic nervous system, resulting in abnormal heart rate and blood pressure. There is no known cause of the Guillain–Barré syndrome, but research suggests that infection is often involved (5). As well as bacterial and viral infections, vaccines and surgery can trigger GBS. There have been reports of an unexpected rise in GBS cases in countries affected by Zika virus infection. On the basis of the available evidence, there is a good possibility that Zika virus infection causes GBS (6). Currently, there is no known drug that can effectively treat GBS; however, supportive care can decrease symptoms and shorten its duration. Supportive care includes monitoring breathing, heartbeat, and blood pressure. The patient is usually placed on a ventilator if their breathing ability is impaired. Early diagnosis is the key to facilitating clinical decision making and treatment selection. Therefore, the aim of the present study was to identify mRNA signatures that may serve as early diagnostic biomarkers for GBS.

## Materials and methods

### Data sources

In total, two transcriptome datasets (GSE31014 and GSE72748) were downloaded from the GEO website^1^ for the current relative transcriptome analysis. Additionally, we collected plasma samples of one patient with the Guillain–Barré syndrome before treatment (before), and at the end of treatments (After). Oral informed consent was obtained from the participant. Treatment and care were designed in accordance with the WHO criteria for the Guillain–Barré syndrome (1).

### Bioinformatics and statistics analyses

Microarray data from GSE31014 was normalized by GC-Robust Multi-array Average (gcRMA) (7). Before analyzing gene expression, all the genes were transformed by a log2 coefficient. Differential gene expression analysis based on gene effect size (ES > 2), and false discovery rate (FDR < 0.05) (8, 9). RNA-Seq data from GSE72748 was processed by the nf-core/rnaseq pipeline (10). The ROC curve and forward search was performed with the R package “MetaIntegrator” (11). All the statistical analyses were performed with R (version 4.1.3). We imputed missing values using the MetImp (12). We obtained the meta-score by subtracting the mean expression of upregulated genes from the mean expression of downregulated genes.

### RNA isolation and RT-PCR analysis

The RNA isolation was achieved by QIAamp RNA Blood Mini Kit according to the manufacturer’s recommendations (Qiagen). cDNA was synthesized using the QuantiTect Reverse Transcription Kit (Qiagen). The qPCR methodology and primers were performed according to published articles (13, 14).

## Results

### Differential gene expression analysis

**FIGURE 1:**
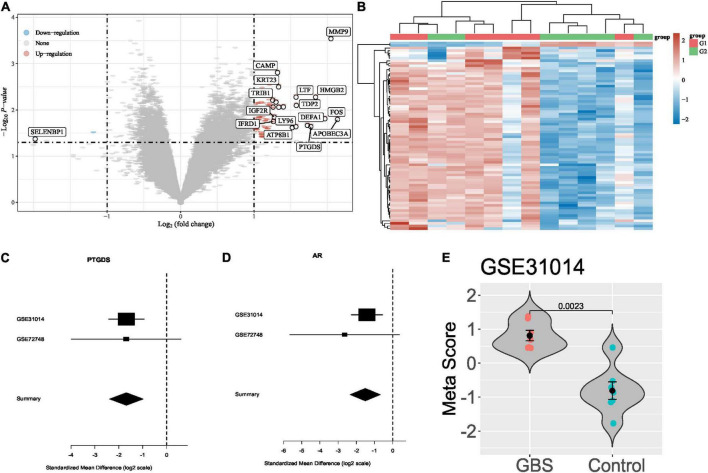
Differential gene expression analysis of GBS. **(A,B)** Differentially expressed genes between GBS and controls in GSE31014; **(C,D)** forest plots of PTGDS and AR gene in GSE31014 and GSE72748; **(E)** violin plot showing the performance of the 2-gene signature for separating GBS from control in GSE31014.

In total, two transcriptome data between GBS and controls were collected from publicly available databases (GEO). Based on the criteria described in methods, we identified differentially expressed genes in Figures 1A,B. After a forward search in the training and validation dataset. In total, two significantly differentially expressed genes (PTGDS and AR), which were down-regulated in the GBS group in both cohorts (Figures 1C,D). Figure 1E shows the meta-scores of each sample in GSE31014. A statistically significant difference was observed between the GBS and control group (*P* < 0.05). Next, we used the two genes to build a GBS diagnostic signature.

### Validation of the two-gene signature in two datasets of Guillain–Barré syndrome

**FIGURE 2:**
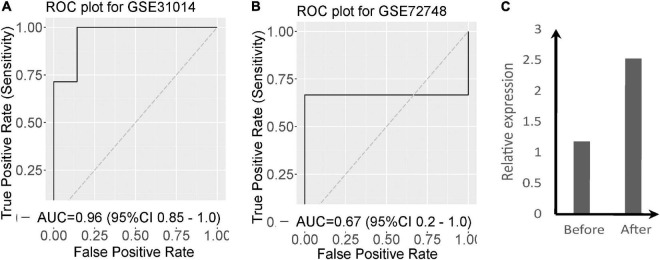
Validation of the two-gene signature in two datasets of GBS. **(A)** ROC curves and AUCs of the two-gene signature classification in GSE31014; **(B)** ROC curves and AUCs of the two-gene signature classification in GSE72748; **(C)** The expression of PTGDS of GBS patients before and after supportive care.

To evaluate the diagnostic power of the 2-gene signature we performed an ROC analysis. The two-gene signature distinguishes GBS from normal with AUC = 0.96 [95% CI 0.85–1] in GSE31014 (Figure 2A). In GSE72748, the two-gene signature distinguishes GBS from normal with AUC = 0.67 (Figure 2B). To investigate the effect of supportive care on the two significant genes, we collected blood samples before supportive care and after supportive care in one patient with GBS. Figure 2C shows that the expression of PDGTS was increased after supportive care.

### Immune cell fractions and immune checkpoint analysis

**FIGURE 3:**
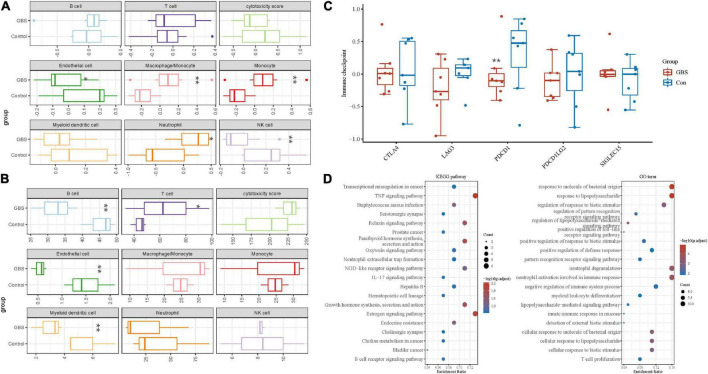
Immune cell fractions and immune checkpoint analysis. **(A,B)** Immune cell fractions in GSE31014 and GSE72748. **(C)** Immune-checkpoint-related genes in the discovery dataset. **(D)** The KEGG pathway and GO term analysis in the discovery dataset. *p < 0.05; **p < 0.01.

Figures 3A,B shows immune cell fractions from two transcriptome data. Both datasets have a significant difference in Endothelial cells. As shown in Figure 3C, PDCD1 immune checkpoint genes are down-regulated and expressed in the GBS group. Figure 3D shows the KEGG pathway classification (left panel), and the right panel shows the 20 most significantly enriched GO terms.

## Discussion

The Guillain–Barré syndrome is a rare neurological disease for which there is no effective treatment. Thus, early diagnosis is a particularly meaningful issue that lets immediate measure toward treatment. The current cytoalbuminologic dissociation method for GBS detection requires an invasive biopsy (15). Therefore, the identification of novel non-invasive biomarkers for GBS diagnosis is preferred. By transcriptome analyses, we identified two new genes (PTGDS and AR) potentially related to GBS. Furthermore, the expression of PTGDS and AR was significantly decreased in the GBS group. Androgen receptor (AR) genes are responsible for constructing an androgen receptor protein. Androgen receptors function as DNA-binding transcription factors that control gene expression (16). During puberty and before birth, androgens play an important role in male sexual development. Prostaglandin-H2 D-isomerase (PTGDS) is an enzyme that converts prostaglandin H2 (PGH2) into prostaglandin D2 (PGD2). As a neuromodulator and trophic factor, PGD2 plays a key role in the central nervous system (17). Moreover, smooth muscle contraction and relaxation are also controlled by PGD2, and platelet aggregation is inhibited by it (18). Interestingly, the expression of this gene increased after the patient was given supportive care. This gene may be a potential target for treating GBS.

## Data availability statement

The original contributions presented in this study are included in the article/supplementary material, further inquiries can be directed to the corresponding author.

## Ethics statement

The studies involving human participants were reviewed and approved by Maternal and Child Health Hospital of Hubei Province. Written informed consent to participate in this study was provided by the participants’ legal guardian/next of kin.

## Author contributions

ZH: study conception. KH: data collection and analysis. KH and ZH: draft manuscript. All the authors reviewed the results and approved the final version of the manuscript.

## Footnotes

1
http://www.ncbi.nlm.nih.gov/geo/

## References

[1] Willison HJ Jacobs BC van Doorn PA. Guillain-barre syndrome. *Lancet.* (2016) 388:717–27. 10.1016/S0140-6736(16)00339-1 26948435

[2] Yuki N Hartung H-P. Guillain–barré syndrome. *N Engl J Med.* (2012) 366:2294–304. 10.1056/NEJMra1114525 22694000

[3] Wijdicks EF Klein CJ. Guillain-barre syndrome. *Mayo Clin Proc.* (2017) 92:467–79. 10.1016/j.mayocp.2016.12.002 28259232

[4] Hughes RA Wijdicks EF Benson E Cornblath DR Hahn AF Meythaler JM Supportive care for patients with Guillain-Barré syndrome. *Arch Neurol.* (2005) 62:1194–8. 10.1001/archneur.62.8.1194 16087757

[5] Allos BM. Association between campylobacter infection and Guillain-Barré syndrome. *J Infect Dis.* (1997) 176:S125–8. 10.1086/513783 9396695

[6] Krauer F Riesen M Reveiz L Oladapo OT Martínez-Vega R Porgo TV Zika virus infection as a cause of congenital brain abnormalities and Guillain–Barré syndrome: systematic review. *PLoS Med.* (2017) 14:e1002203. 10.1371/journal.pmed.1002203 28045901 PMC5207634

[7] Wu Z Irizarry RA. Preprocessing of oligonucleotide array data. *Nat Biotechnol.* (2004) 22:656–8. 10.1038/nbt0604-656b 15175677

[8] Henmi M Copas JB. Confidence intervals for random effects meta-analysis and robustness to publication bias. *Stat Med.* (2010) 29:2969–83. 10.1002/sim.4029 20963748

[9] Enzmann DJJ. *Notes On Effect Size Measures For The Difference Of Means From Two Independent Groups: The Case Of Cohen’sd And Hedges’g January 12, 2015*. (2015).

[10] Ewels PA Peltzer A Fillinger S Patel H Alneberg J Wilm A The nf-core framework for community-curated bioinformatics pipelines. *Nat Biotechnol.* (2020) 38:276–8. 10.1038/s41587-020-0439-x 32055031

[11] Haynes WA Vallania F Liu C Bongen E Tomczak A Andres-Terrè M Empowering multi-cohort gene expression analysis to increase reproducibility. *Proceedings of the Pacific Symposium on Biocomputing 2017.* Singapore: World Scientific (2017). p. 144–53. 10.1142/9789813207813_0015 PMC5167529 27896970

[12] Wei R Wang J Su M Jia E Chen S Chen T Missing value imputation approach for mass spectrometry-based metabolomics data. *Sci Rep.* (2018) 8:663. 10.1038/s41598-017-19120-0 29330539 PMC5766532

[13] Ren Z Gao M Jiang W. Prognostic role of NLGN2 and PTGDS in medulloblastoma based on gene expression omnibus. *Am J Trans Res.* (2022) 14:3769. PMC9274574 35836891

[14] Catteau-Jonard S Jamin SP Leclerc A Gonzalès J Dewailly D Di Clemente N. Anti-Mullerian hormone, its receptor, FSH receptor, and androgen receptor genes are overexpressed by granulosa cells from stimulated follicles in women with polycystic ovary syndrome. *J Clin Endocrinol Metab.* (2008) 93:4456–61. 10.1210/jc.2008-1231 18697861

[15] Van der Meché F Van Doorn P. Guillain-Barre syndrome and chronic inflammatory demyelinating polyneuropathy: immune mechanisms and update on current therapies. *Ann Neurol.* (1995) 37:14–31. 10.1002/ana.410370704 8968214

[16] Gelmann EP. Molecular biology of the androgen receptor. *J Clin Oncol.* (2002) 20:3001–15. 10.1200/JCO.2002.10.018 12089231

[17] Zhao W Jiang B Hu H Zhang S Lv S Yuan J Lack of CUL4B leads to increased abundance of GFAP-positive cells that is mediated by PTGDS in mouse brain. *Hum Mol Genet.* (2015) 24:4686–97. 10.1093/hmg/ddv200 26025376

[18] Rezaee S Kakavandi N Shabani M Khosravi M Hosseini-Fard SR Najafi M. COX and PTGDS gene expression levels in PGD2 synthesis pathway are correlated with miR-520 in patients with vessel restenosis. *Endocr Metab Immune Disord Drug Targets.* (2020) 20:1514–22. 10.2174/1871530320666200511012142 32389118

